# The impact of coordination-based movement education model on balance development of 5-year-old children

**DOI:** 10.3389/fpsyg.2022.1045155

**Published:** 2023-01-04

**Authors:** Hüseyin Tolga Esen, Aziz Güçlüöver, Mert Kurnaz, Mustafa Altinkök

**Affiliations:** ^1^Department of Physical Education and Sports, Faculty of Sport Sciences, Akdeniz University, Antalya, Turkey; ^2^Department of Physical Education and Sports Education, Faculty of Sports Sciences, Kırıkkale University, Kırıkkale, Turkey; ^3^Department of Physical Education and Sport, School of Physical Education and Sport, Haliç University, İstanbul, Turkey

**Keywords:** education method, movement education, balance, coordination, early childhood

## Abstract

With this study, it was aimed to examine the effect of coordination-based movement education model on the development of balance in 5-year-old children. The research was designed with a control group pre-test post-test design, which is one of the quasi-experimental research models. The research group consists of a total of 42 (*n* = 20 experimental *n* = 22 control) 5-year-old children formed by using the convenient sampling method, which is one of the purposeful sampling methods. Each participant’s age, body weight, body length and lower extremity limb lengths were measured. As a data collection tool and used the three-part Y Balance Test (YBT) platform, which was previously stated to have high reliability (ICC = 0.85–0.93). Reach distances of the participants were measured in three directions, anterior posteromedial and posterolateral. YBT scores were determined by calculating the average and normalized values for lower extremity limb length for each reach direction of the obtained scores, and composite YBT scores were determined by taking the averages of anterior, posteromedial and posterolateral reach distance scores. In order to determine whether the collected data are suitable for normal distribution, Levene test was applied first and it was determined that *p* > 0.05 for all parameters as a result of the test. Independent sample *T*-test from parametric tests was used to determine the differences between groups. Paired-group *T*-test was used to determine within-group differences. According to the results of the research, it was found that the balance motor capacity levels between the experimental and control groups did not differ significantly compared to the pre-test, but there was a significant difference in all reach directions scores in favor of the post-test and experimental group compared to the control group and the pre-test. When the results of the right and left lower extremity ANT, PM, PL and Composite reach distances were examined according to gender, although there was no statistically significant difference, when the averages were considered, it was seen that all parameters were in favor of girls (*p* > 0.05). As a result, it was concluded that coordination-based movement education model had a positive effect on the balance motor capacities of 5-year-old children.

## 1. Introduction

In the early childhood period, movement and mobility are a part of the child’s life, and it is known that with the birth, children begin to discover their own bodies, abilities and their environment with both coarse and fine muscular, voluntary or involuntary movements. It’s been noted worldwide that the majority of the children who are at the preschool stage not encouraged to engage in sufficient movement and physical activity practices, if at all, and the COVID-19 period has led to far more excessive sedentary habits among preschool children as a result of COVID restrictions or the risk of getting infected (Chaput et al., 2016, 2017; Berglind and Tynelius, 2017; Santos et al., 2017; De Craemer et al., 2018). According to the World Health Organization (WHO) engagement in preschool implementations based on enough physical activity and exercise will reduce sedentary lifestyle, especially in the current COVID-19 pandemic period, and will be the key to positive health outcomes in both short and long term (World Health Organization, 2019). Rosário (2014) defines posture as the orientation of the body in specific positions. It can be described in both stillness and movement. The ability to control one’s body position in space for the purposes of movement and balance is referred to as postural stability (Woollacott and Shumway-Cook, 2002). It is required for maintaining a static position as well as assisting with body coordination during dynamic position changes. Musculoskeletal disorders can be caused by prolonged poor or incorrect posture. Gallahue (1996) emphasizes that developmental fundamental movement education plays a significant role to contribute the development of motor movement, motor performance and motor skills. It is emphasized that at the preschool stage, the fundamental motor skills which are the building blocks for more complex motor skills to be involved in games as well as fundamental movement skill education, play a significant role in children’s development of specific motor activity and develop through exercise along with physical maturation (Hardy et al., 2012; Logan et al., 2018; Gallahue et al., 2019). As a result, the preschool stage is regarded as a critical period for the development of fundamental movement skills, postural balance and postural control. Also, it is claimed that fundamental motor skill performance can help in daily life and facilitate the participation in sports activities at a later age, as well as contribute positively to increasing health, postural balance, postural control and academic achievement (Stodden et al., 2008; Jaakkola et al., 2015). Therefore, it is stated that developmental fundamental movement education practices should be a part of the physical education program starting from the preschool stage until the university, and that participation in these practices contributes positively to the development of certain fundamental motor movement skills and performances (Cooper et al., 1989). One of these fundamental motor movement performance capacities is balance capacity.

In general, balance is defined in two ways as: static (stationary) and dynamic (moving). Static balance refers to the capacity to maintain balance in a fixed position, while dynamic balance refers to the capacity to maintain the balance by maintaining a stable position during moving positions that require reaching or walking (Pollock et al., 2000). It is emphasized that balance performance capacity is a vital capacity that is considered necessary throughout life, particularly during the preschool stage, as well as a very important factor in controlling mobility and coordinated movements (Ringhof and Stein, 2018), forming our daily basic movements, and performing various movement forms (Huxham et al., 2001). Balance performance development is an important factor that contributes to children’s other motor performance capacity and skill development, particularly in early childhood (Huxham et al., 2001). Furthermore, it is stated that poor balance performance in the preschool phase increases the risk of handicap or injury (Ringhof and Stein, 2018).

Balance skill development is known to be important and critical, especially in the early childhood stage. Furthermore, there are gender differences in the development of static and dynamic balance skills in children at this stage. There are also conflicting findings regarding gender differences in children’s static and dynamic balance skills in early childhood (Li et al., 2022). According to Kakebeeke et al. (2012) and Latorre-Román et al. (2021), there was no significant difference in static and dynamic balance skills in early childhood between boys and girls. Girls outperform boys in static and dynamic balance skills, according to DeOreo and Wade (1971), Lee and Lin (2007), Cadenas-Sanchez et al. (2019), Shams et al. (2020) and Altınkök (2021). It is seen that there is limited research on the development of static and dynamic balance skills in early childhood. Although the findings of these studies indicate that static and dynamic balance skills develop with age, more research is required to investigate gender differences in static and balance skills of children in the early childhood stage. It is claimed that there is a significant correlation in both movement and physical activity, musculoskeletal, cardiorespiratory, cardiovascular, cardiometabolic health, and motor and cognitive skill profiles in children in early childhood (Carson et al., 2019).

It is thought that there are limited resources and more research is needed to evaluate the effects of fundamental movement education model on fundamental motor movement performance and skills for children in early childhood. It was hypothesized that the static and dynamic balance increased with age and that there was a difference according to being in the experimental group and gender. In addition, it is hypothesized that there is a significant difference in the static and dynamic balance levels of girls compared to boys. In this context, with this research, it is aimed to determine the effect of coordination-based movement education model on the balance motor performance development of 5-year-old children.

## 2. Materials and methods

### 2.1. Research design and experimental approach to the problem

This research, which aims to reveal the effect of coordination-based movement education model carried out under the control of researchers on the development of balance motor performance capacities, was designed with a control group pre-test post-test design, which is one of the quasi-experimental research models. While the research group was limited, the convenience sampling method, which is one of the purposeful sampling methods, was used in order to prefer 5-year-old preschool children who are in an easily accessible preschool education institution, have no attendance problems, have high participation in classes and have no barriers to participation in the practices. Five-year-old preschool children with similar backgrounds and socioeconomic levels were determined, and the experimental and control groups were formed by objective assignment, in order to eliminate the factors that may adversely affect the internal validity in the implementations and maintenance of the applications and in the procedures related to the application, and to minimize the defects that may occur in the research.

### 2.2. Research group and participants

Demographic information and informed consent form were obtained from the parents for each participant before the coordination-based movement education model, those with complaints of lower extremity and spine pain or medical or neuromuscular-skeletal system disorders that limit participation in exercise and those who refused to participate in the exercise were not included in the study. The research study group consisted of 42 five-year-old preschool students (*n* = 20 experiment *n* = 22 control) enrolled in preschool education institutions who met the specified criteria and had no school attendance problems. Furthermore, all the procedures of the study were approved by the ethics committee.

Table 1 provides the information of the demographic characteristics and average values of the participants.

**Table 1:** Demographic characteristics and frequency percentage distributions and average values of the 5-year-old preschool children participating in the study.

	Experimental (*n* = 20% 47.6)	Control (*n* = 22% 52.4)	*N*	%
Gender (f)	9	12	21	50.0
Gender (m)	11	10	21	50.0
Mass (kg)	22.48 **±** 4.88	24.31 **±** 5.75		
Height (cm)	1.17**±** 0.052	1.20**±** 0.066		
Lower limb length (cm)	68.60 **±** 3.36	70.59 **±** 4.89		

x̄ ± SS, mean-standart deviation; cm, centimeter; kg, kilogram; *n*, number; %, percentage; f, female; m, male.

### 2.3. Data collection tool

#### 2.3.1. Y balance test

The Y Balance Test requires a balanced stance with one lower extremity limb while reaching the anterior (ANT), posterolateral (PL) and posteromedial (PM) directions on the test instrument. With the Y Balance Test, it is aimed to determine the dynamic balance performance capacity levels of the participants while they are moving in three directions (ANT, PM, PL) with each lower extremity limb during stance (Plisky et al., 2009).

In the study, Y Balance Test was used as a Y balance platform (Shaffer et al., 2013), which was previously stated to have high reliability (ICC = 0.85–0.93) and consists of three parts (Move2-Perform, Evansville, Indiana, United States; Park et al., 2020).

##### 2.3.1.1. Implementation of Y balance test

Plisky et al. (2006) provide a brief instructional demonstration to the participants, demonstrating the protocol and application of the test as indicated. Furthermore, Earl and Hertel (2001) stated that the longest reach on the Y Balance Test platform was achieved after six trials in each direction. As a consequence, before the actual test evaluations, the participating children were allowed to perform six trials on each of the three access directions and each lower extremity (Engquist et al., 2015). All of the children who took part in the trials were evaluated for the actual test about 20 min after the trials and did not wear sneakers during the process of data collection. Participants were instructed to stand on one leg, toes behind the starting line, and to reach their free lower extremities in the ANT, PM, and PL directions according to their stance legs while maintaining a one-leg stance. A standard test sequence has been established to improve test reproducibility and to create a consistent test protocol. The established test sequence was repeated by reaching the right foot three times in the ANT direction, then the left foot three times in the ANT direction, and the same procedure was repeated for the PM and PL directions (Plisky et al., 2009).

In the actual test evaluations, the participant children were instructed by the researchers to stand on one foot on the test platform with the toes behind the starting line, then reach the tested direction at the target distance without touching the floor, and then return to the starting position while maintaining the same position This is the only instruction given to the children who are taking part in the test. According to the criteria determined in the six main test evaluations, it was noted that the participant children failed in whichever direction they could not perform the test (Plisky et al., 2009).

The actual test assessments have been canceled and repeated if:

- When standing on one leg cannot be maintained on the platform. For example, touching the floor with the reach foot or falling off the platform.
- When there is a pause in the movement pattern while moving and reaching in the target direction with the reach limb. Exemplifications include reaching and retracting the reach limb or failing to maintain balance within the maximum reach distance.
- When the reach limb cannot be set to the start position in a controlled manner.

##### 2.3.1.2. Lower extremity limb length measurement

After the participants lying on their back on a mat, the researchers measured the length of the lower extremity in centimeters with a meter gauge from the anterior superior iliac to the most distal part of the medial malleolus (Plisky et al., 2009).

##### 2.3.1.3. Evaluation of Y balance test results

The results of the actual test evaluations were analyzed based on each participant’s lower extremity length in the ANT, PL, and PM reach directions. Means and standard deviations were calculated for reach distances and lower extremity lengths in the ANT, PL, and PM directions, and because reach distance is related to lower extremity limb length, the reach distance was normalized to lower extremity limb length. The normalized value was calculated by dividing the reach distance by the lower limb length and multiplying by 100 to express the reach distance as a percentage of the lower limb length. The composite reach distance (average of the three reach directions) was obtained by dividing the sum of the three reach directions by three times the lower extremity limb length and then multiplying by 100 (Gribble and Hertel, 2004; Plisky et al., 2006).

### 2.4. The data collection process

Measurement and evaluations were made by two researchers who recorded the scores obtained from the Y Balance Test based on coordination-based movement education model. The data collection process was carried out in three phases and is as follows.

#### 2.4.1. Preliminary studies and pre-test implementation

A preliminary study was conducted prior to beginning the 10 week coordination-based movement education model to start the pre-test, apply the Y Balance Test instructions, organize the Y Balance Test measurement platform, calculate the average time spent per Y Balance Test, and test the Y Balance Test measurement platform. Following the completion of the preliminary studies, the Y Balance Test pre-tests were completed in order to determine the balance level, with one working day separate for the experimental and control groups. All of the preliminary studies and applications were made by the researchers.

#### 2.4.2. Learning–teaching processes

The coordination-based movement education model, which was used in the research process and whose effects on balance performance were investigated, was developed and applied by the researchers under the supervision of experts and in accordance with the developmental characteristics of the age group. Following the pre-studies and pre-test measurements, the children in the control group continued their desk-based standard preschool education. On the other hand, coordination-based movement education model were applied to the experimental group participants, which facilitated the development of balance performance and variations, as well as the development of coordinated movement performances with parkour, games, and stations designed one after the other. For a total of 10 weeks, the children in the experimental group actively participated in coordination-based movement education model implementations, two different days a week and 40-min moderate-intensity movement activities per day. The activities were carried out at the school where the children are registered and during the school’s movement and play class hours.

Table 2 shows the activities and durations applied in the study. We administered the post-test, which we had applied to the students in the experiment group and control group at the beginning of the movement program, again at the end of 10 weeks.

**Table 2:** Exercises with the experimental group and their duration.

	Activities by week	1.	2.	3.	4.	5.	6.	7.	8.	9.	10.
	1. Exercises on the mat	X	X	X	X			X		X	X
	2. Rope movements		X	X			X		X		X
	3. Movements in balance equipment				X		X		X	X	X
	4. Movements in the ground balance equipment					X	X			X	X
	5. Paired movements						X	X		X	X
	6. Rhythm and coordination movements							X	X	X	X
	7. Increase muscular strength and endurance	X	X		X	X		X		X	X
	8. Non dominant eye or hand activities and stepping stones		X	X			X		X	X	
	9. Postural control skills	X	X	X	X	X	X	X	X	X	X

The exercise time was limited to 40 min. Before starting each activity preparation and warm-up activities, which lasted about 5 min, were applied to the experimental group during the preparation phase of the lessons. In the main phase of the lesson, which lasted approximately 30 min, implemented to containing of development children’s balance, coordination, agility, flexibility, repetition of the previous practice and new fundamental movement skills the coordination-based movement education model in accordance with the parameters specified in Table 2. In the finishing phase of the lessons, cooling and relaxation activities, which lasted about 5 min, were applied. In the implementation of the coordination-based movement education model, all other activities in the preschool were the same between the experimental and control groups, except for the content of the model and the different hours of the lessons. During the implementation of the model, the experimental group did not attend any other physical education classes.

#### 2.4.3. Post-test implementation

At the end of 10 weeks, in order to reveal the effect of coordination-based movement education model on balance performance capacity, the Y Balance Test was applied to the experimental and control groups by following the procedure applied in the pre-test in the 11th week.

### 2.5. Statistical analyses

The results were statistically analyzed using the SPSS 22.0 software. In order to determine whether the collected data are suitable for normal distribution, Levene test was applied first and it was determined that *p* > 0.05 for all parameters as a result of the test. Then, independent sample T test from parametric tests was used to determine the differences between groups. Paired-group *T*-test was used to determine within-group differences. Frequency percentage distributions, body length, body weight, lower extremity limb length, mean and standard deviation values of ANT, PM, PL and Composite reach distance values for all participants’ gender and group (experiment-control) variables were analyzed and interpreted. Cohen’s d method was used to calculate the effect size (Cohen, 1992). In the study, the confidence interval was determined as 95% and the significance level was determined as *p* < 0.05.

## 3. Results

As shown in Table 3, the results of the independent sample T-test, which was conducted to compare the ANT, PM, PL and Composite reach distance scores of the Y Balance Test post-test measurements, based on coordination-based movement education model implementations between the experimental and control groups; It is seen that there is a statistically significant difference in favor of the post-test and experimental group in the mean values of the right and left lower extremities in ANT, PM, PL and Compsoite reach distances compared to the control group (*p* < 0.05). It is assumed that the finding of a significant difference in favor of the post-test and experimental group is due to the implementation of coordination-based movement education model.

**Table 3:** The results of the independent sample *T*-test, which was conducted to determine whether there is a significant difference between the balance motor performance capacity post-test Y Balance Test ANT, PM, PL and composite reach distance mean scores of the experimental and control groups.

	Experimental (*n* = 20)	Control (*n* = 22)	t	%	*p*	d
**Anterior reach distance**						
Right lower extremity	94.94 ± 11.74	82.67 ± 24.23	2.053	18.37	0.047^*^	−0.644
Left lower extremity	97.86 ± 24.71	79.81 ± 20.19	2.601	22.62	0.013^*^	−0.799
**Posteromedial reach distance**						
Right lower extremity	96.51 ± 10.28	77.78 ± 29.31	2.708	24.08	0.010^*^	−0.852
Left lower extremity	95.37 ± 26.82	72.88 ± 26.16	2.748	30.87	0.009^*^	−0.848
**Posterolateral reach distance**						
Right lower extremity	97.93 ± 11.39	81.95 ± 28.31	2.355	19.50	0.024^*^	−0.740
Left lower extremity	96.68 ± 26.04	78.65 ± 25.68	2.257	25.47	0.030^*^	−0.697
**Composite reach distance**						
Right lower extremity	87.82 ± 30.94	78.76 ± 25.21	0.814	11.50	0.049^*^	−0.321
Left lower extremity	98.58 ± 21.13	75.55 ± 23.36	0.337	30.48	0.002^*^	−1.033

x̄ ± SS, mean-standard deviation; *n*, number; *t*, *t*-test score; %, percentage of difference; d, cohen’s d value.

* *p* < 0.05.

As shown in Table 4, the results of the paired group *T*-test, which was conducted to compare the ANT, PM, PL and Composite reach distance scores of the Y Balance Test pre–post measurements, based on coordination-based movement education model implementations the experimental group; It is seen that there is a statistically significant difference in favor of the post-test and in the mean values of the right and left lower extremities in ANT, PM and PL reach distances compared to the pre-test (*p* < 0.05, *p* < 0.01). It is assumed that the finding of a significant difference in favor of the post-test is due to the implementation of coordination-based movement education model.

**Table 4:** Paired group *T*-test results conducted to determine whether there is a significant difference between the experimental group balance motor performance capacity pre–post-test Y Balance Test ANT, PM, PL and Composite reach distance mean scores.

	Pre-test	Post-test	t	%	*p*	d
**Anterior reach distance**						
Right lower extremity	82.50 ± 24.49	94.94 ± 11.74	−2.108	15.07	0.049^*^	−0.647
Left lower extremity	80.01 ± 22.42	97.86 ± 24.71	−5.666	22.30	0.000^**^	−0.756
**Posteromedial reach distance**						
Right lower extremity	80.69 ± 23.58	96.51 ± 10.28	2.825	19.60	0.011^*^	−0.869
Left lower extremity	77.54 ± 26.01	95.37 ± 26.82	−4.995	22.99	0.000^**^	−0.674
**Posterolateral reach distance**						
Right lower extremity	86.74 ± 22.19	97.93 ± 11.39	−2.108	12.90	0.049^*^	−0.634
Left lower extremity	83.85 ± 28.74	96.68 ± 26.04	−2.216	15.30	0.039^*^	−0.467
**Composite reach distance**						
Right lower extremity	70.56 ± 16.98	87.82 ± 30.94	−2.170	24.46	0.043^*^	−0.691
Left lower extremity	76.76 ± 22.96	98.58 ± 21.13	7.094	26.77	0.000^**^	−0.988

x̄ ± SS, mean-standard deviation; *n*, number; *t*, *t*-test score; %, percentage of development; d, cohen’s d value.

* *p* < 0.05.

** *p* < 0.01.

As shown in Table 5, the results of the independent sample T-test, which was conducted to compare the ANT, PM, PL and Composite reach distance scores of the Y Balance Test post-test measurements, based on coordination-based movement education model implementations between gender; Although there was no statistically significant difference in ANT, PM, PL and Composite reach distances in the right and left lower extremities when the averages are considered, it is seen that all parameters are in favor of girls (*p* > 0.05).

**Table 5:** The results of the independent sample *T*-test, which was conducted to determine whether there is a significant difference between the balance motor performance capacity post-test Y Balance Test ANT, PM, PL and Composite reach distance mean scores of gender.

	Girls (*n* = 21)	Boys (*n* = 21)	t	%	*p*
**Anterior reach distance**					
Right lower extremity	90.11 ± 18.60	86.91 ± 21.79	0.511	3.68	0.612
Left lower extremity	90.46 ± 24.83	86.35 ± 23.53	0.550	4.76	0.585
**Posteromedial reach distance**					
Right lower extremity	90.34 ± 13.15	83.06 ± 31.39	0.981	8.76	0.332
Left lower extremity	89.51 ± 26.49	77.67 ± 29.85	1.360	15.24	0.181
**Posterolateral reach distance**					
Right lower extremity	92.49 ± 22.40	86.62 ± 24.06	0.818	6.78	0.418
Left lower extremity	89.27 ± 25.50	85.20 ± 29.12	0.481	4.78	0.633
**Composite reach distance**					
Right lower extremity	85.47 ± 27.66	82.78 ± 28.89	0.309	3.25	0.759
Left lower extremity	90.45 ± 21.00	82.59 ± 28.30	1.022	9.52	0.313

x̄ ± SS, mean-standard deviation; *n*, number; *t*, *t*-test score; %, percentage of difference.

As seen in Table 6, paired samples *t*-test results for comparing ANT, PM, PL and Composite reach distance scores of Y Balance Test pre–post-test measurements based on coordination-based movement training model applications were among the control group; it is seen that there is no statistically significant difference in ANT, PM, PL and Composite reach distances in the right and left lower extremities (*p* > 0.05). Based on this finding, it is thought that the standard desk-based preschool education program has little effect on balance skills.

**Table 6:** Paired group *T*-test results conducted to determine whether there is a significant difference between the control group balance motor performance capacity pre–post-test Y Balance Test ANT, PM, PL and Composite reach distance mean scores.

	Pre-test	Post-test	t	%	*p*	d
**Anterior reach distance**						
Right lower extremity	77.61 ± 21.64	82.67 ± 24.23	−1.267	6.52	0.219	−0.220
Left lower extremity	78.49 ± 23.93	79.81 ± 20.19	−0.302	1.68	0.766	−0.059
**Posteromedial reach distance**						
Right lower extremity	72.75 ± 27.98	77.78 ± 29.31	−1.293	6.91	0.210	−0.175
Left lower extremity	70.01 ± 21.48	72.88 ± 26.16	−0.533	4.10	0.599	−0.119
**Posterolateral reach distance**						
Right lower extremity	82.53 ± 27.33	81.95 ± 28.31	0.083	−0.70	0.934	0.020
Left lower extremity	76.65 ± 31.99	78.65 ± 25.68	−0.330	2.61	0.745	−0.068
**Composite reach distance**						
Right lower extremity	77.63 ± 22.95	78.76 ± 25.21	−0.789	1.46	0.439	−0.046
Left lower extremity	73.47 ± 23.39	75.55 ± 23.36	−0.461	2.84	0.650	−0.088

x̄ ± SS, mean-standard deviation; *t*, *t*-test score; %, percentage of development; d, cohen’s d value.

## 4. Discussion, conclusion, and recommendations

This current research aims to examine the effect of coordination-based movement education model on the development of balance performance capacities in 5-year-old children.

Our research hypothesis that girls generally perform better than boys in balance skills, which is one of the gross motor skills, and that they are in the experimental group in the early childhood stage was partially confirmed. Although there is no significant difference according to the results of the comparison according to gender, when the averages are considered, it is seen that girls are in favor of all parameters. According to the comparison results made according to being in the experimental group, it is seen that there is a statistically significant difference in favor of the experimental group and post-tests in all parameters. The findings show that gender should be taken into account when determining balance skills in early childhood children. These findings can guide health, physical education and school professionals in using multiple methods to identify early childhood children with high or very low balance skills and to design appropriate movement tasks for boys and girls.

In the research, coordination-based movement education model was applied to the experimental group for a total of 10 weeks, 2 days a week, and 40 min per lesson, in the preschool program’s movement activities classes, which included movement and game activities. The control group, on the other hand, continued in the standard desk-based preschool lessons and were not included in the coordination-based movement education model.

When the literature is examined, very limited study the preschool stage is found that evaluates the relationship between Y Balance Test performance and movement, play, or physical activity. Nevertheless, studies show that practices involving movement, play, or physical activity improve balance (Barrett and Smerdely, 2002; Costa et al., 2009; Overmoyer and Reiser, 2015; Plazibat et al., 2021; Tiktampanidi et al., 2021). It has also been reported that resistance and flexibility exercises help to improve balance performance (Barrett and Smerdely, 2002). There are numerous tests that can be used to measure the level of balance. The Y Balance Test is one of these tests. The Y balance test performance of 188 children in the first and fifth grades was evaluated by the researchers. The study concluded that test–retest reliability scores remained consistent for 7–10 days and that typical error values for all access directions and grade levels were less than 10% of the mean (Labella et al., 2014). In line with the aim of the research, The Y Balance Test (Thorpe and Ebersole, 2008), a dynamic balance test used to provide an accurate interpretation and measurement value for the neuromuscular and coordinated control of the lower extremities and limbs, was used. Furthermore, based on the research’s main findings. It was concluded that coordination-based movement education model contributed to the positive development of balance motor performance capacities in 5-year-old children who received movement activity. According to the literature, higher Y Balance Test scores indicate better dynamic balance (Park et al., 2020). A study that examined the physical fitness of children living in urban areas of the United States found that children whose physical activity levels should have been maintained at a high level but were not, experienced cardiovascular problems. As a result, it has been concluded that it is critical to pay attention to movement education models developed in early childhood and childhood. Furthermore, it has been reported that children who participate in early childhood stage movement education models will increase their physical activity, development, and movement skills by collaborating with experts in this field (Washington, 2001). Gymnastics and movement training interventions, according to Fotiadou et al. (2002), improve static and dynamic balance capacity in preschool children. In addition, it is claimed that girls who participate in activities such as dance and gymnastics more often than boys contribute to the development of balance skills (Jing et al., 2019). Dance programs, according to Kostic et al. (2003), can also improve balance capacity, side walking, and backward walking on a platform. Furthermore, Zivcic et al. (2008) concluded that physical activity improves backward walking.

Girls are reported to be better at balance movement than boys until about 7–8 years old (Mckenzie et al., 2002; Spodek and Saracho, 2006), and girls are generally better at dynamic and static balance motor capacities. (DeOreo, 1971; Frederick, 1977; Gallahue and Ozmun, 2006; Spodek and Saracho, 2006). This can be attributed to several possible factors, including earlier maturation of central nervous structures that enable sensory integration and the use of more complex postural control strategies (De Bellis et al., 2001; Peterson et al., 2006; Plandowska et al., 2019). Everke (2009), dynamic balance improved dramatically in backward walking tests at the age of four, with gender differences favoring females. Furthermore, Alpert et al. (1990) proposed that aerobic activities and forward walking on a balance beam increased balance skill. According to a study conducted among children playing volleyball and not having a sportive branch, it was concluded that the balance performance scores of the posterolateral and composite reach directions of the right and left lower extremities were in favor of the volleyball playing group (Ateş, 2017). In a study on women playing football, women football players who play for performance play have a higher lower extremity limb strength than women who do not play performance football, where the results of Star Excursion Balance Test vary by their age of training. It has been reported that they have achieved a significantly greater distance of reach in anterior and posterolateral reach directions, and this may be due to greater neuromuscular control, depending on training and practices involving movement and coordination (Thorpe and Ebersole, 2008). According to the findings of a study that discovered a statistically significant difference in the posteromedial and posterolateral reach aspects of the Y Balance Test but not in the anterior reach direction, balance training practices involving movement and physical activity were applied to the functional test and a lower extremity limb in order to improve the balance variations of healthy individuals (Haksever et al., 2007). This is emphasized that it improves the performance of sustaining the stabilization and makes a positive contribution (Haksever et al., 2007). In a study on women, it was determined that stretching exercises applied for 15 s had a positive effect on dynamic balance performance (Costa et al., 2009). In a study conducted on university students who are athletes, depending on the average reach of the Y Balance Test (Lehr et al., 2013) and on basketball players based on the Star Excursion Balance Test (Plisky et al., 2006) was emphasized that the obtained findings can be used to make predictions about lower extremity disability and injuries that may occur in the future. Furthermore, studies show that the Y Balance Test contributes to the prediction of disability and injuries due to general reach performance and asymmetry between lower extremity limbs (Butler et al., 2013; Lehr et al., 2013; O’Malley et al., 2014).

According to the relevant literature and research results, it is seen that the coordination-based movement education model developed the balance performance capacity of 5-year-old children in the preschool period and is also very effective in the development and excelling of the balance skills of the learning–teaching processes.

Consequently, it was concluded that coordination-based movement education model contributed to the positive development of balance motor performance capacities in 5-year-old children who received movement activity.

More active participation of children in activities should be supported, and effective and efficient learning–teaching processes should be supported in research on preschool children. In addition, modern and learner-centered teaching methods that encourage children’s versatile development should be applied, and it is recommended to increase the number of studies that support the development of other fundamental motor performance capacities, especially balance motor performance capacity. Finally, it is recommended that this model be applied to all ages in the early childhood stage.

### 4.1. Strengths and limitations of the research

The study’s strength is that it is the first in Turkey to investigate dynamic balance skills with the Y Balance Test in children in early childhood. Furthermore, dynamic balance was determined using the Y Balance Test, an international test tool that provides insight into dynamic balance components while increasing the validity and reliability of the results. In addition, the fact that this model is a model that improves dynamic balance capacity is seen as one of the strengths of the research.

Given the study’s limitations, such as the small sample size and geographical differences in Turkey, the sample may not be representative of the country. It is also believed that our findings should be interpreted in a variety of contexts. Finally, the fact that we do not take into account variables such as family income and kindergarten parameters that may affect dynamic balance skills can be viewed as limiting. The research is limited to 5-year-old children in the early childhood stage and the coordination-based movement education model applied in the research.

## Data availability statement

The original contributions presented in the study are included in the article/supplementary material, further inquiries can be directed to the corresponding author.

## Ethics statement

Written informed consent was obtained from the minor(s)’ legal guardian/next of kin for the publication of any potentially identifiable images or data included in this article.

## Author contributions

MA: supervision and conceptualization. MK and MA: intervention, data collection, writing and revision writing. HE: paper screening. AG: statistical analysis. All authors have read and accepted the published version of the article.

## Conflict of interest

The authors declare that the research was conducted in the absence of any commercial or financial relationships that could be construed as a potential conflict of interest.

## Publisher’s note

All claims expressed in this article are solely those of the authors and do not necessarily represent those of their affiliated organizations, or those of the publisher, the editors and the reviewers. Any product that may be evaluated in this article, or claim that may be made by its manufacturer, is not guaranteed or endorsed by the publisher.
